# Exercise upregulates salivary amylase in humans (Review)

**DOI:** 10.3892/etm.2014.1497

**Published:** 2014-01-23

**Authors:** ERI KOIBUCHI, YOSHIO SUZUKI

**Affiliations:** Laboratory of Sports Nutrition and Biochemistry, Graduate School of Health and Sports Science, Juntendo University, Inzai, Chiba 270-1695, Japan

**Keywords:** physical stress, sympathetic nervous system, adrenergic, athlete

## Abstract

The secretion of salivary α-amylase is influenced by adrenergic regulation of the sympathetic nervous system and the hypothalamic-pituitary-adrenal axis; thus, exercise affects the levels of salivary α-amylase. Granger *et al* published a review in 2007 that focused attention on salivary α-amylase. In addition, a portable system for monitoring salivary α-amylase activity was launched in Japan at the end of 2005. The correlation between exercise and salivary α-amylase has since been extensively investigated. The present review summarizes relevant studies published in the English and Japanese literature after 2006. A search of the PubMed and CiNii databases identified 54 articles, from which 15 original articles were selected. The findings described in these publications indicate that exercise consistently increases mean salivary α-amylase activities and concentrations, particularly at an intensity of >70% VO_2_max in healthy young individuals. Thus, these studies have confirmed that salivary α-amylase levels markedly increase in response to physical stress. Salivary α-amylase levels may therefore serve as an effective indicator in the non-invasive assessment of physical stress.

## 1. Introduction

Salivary α-amylase secretion is influenced by adrenergic regulation of the sympathetic nervous system and the hypothalamic-pituitary-adrenal axis (1). Therefore, exercise may affect salivary α-amylase levels.

Granger *et al* (1) published a review of biobehavioral studies of salivary α-amylase in 2007, suggesting that salivary α-amylase levels markedly increase in response to physical and psychological stress. Studies by Chatterton *et al* (2) and Kivlighan and Granger (3) identified that salivary α-amylase levels increased in response to exercise. Chatterton *et al* (2) compared levels of salivary α-amylase in males prior to and following exercise, a written examination or rest, and identified that aerobic exercise induced a three-fold mean increase in α-amylase levels. Kivlighan and Granger (3) observed that salivary α-amylase levels increased by an average of 156% in 42 members (21 females) of a collegiate crew team in response to an ergometer competition.

Following publication of the review by Granger *et al* (1), various groups investigated the correlation between exercise and salivary α-amylase. A portable system for monitoring salivary α-amylase activity was launched in Japan at the end of 2005 (4), which stimulated increased interest in the subject. Certain findings were only published in Japanese. The present review aims to summarize previous studies concerning the correlation between exercise and salivary α-amylase levels published in the English and Japanese literature.

## 2. Materials and methods

Information was collected from the PubMed (http://www.ncbi.nlm.nih.gov/pubmed/) and CiNii (http://ci.nii.ac.jp/) databases. The latter is a database maintained by the Japanese National Institute of Informatics (Tokyo, Japan), which comprises literature published by Japanese authors in academic journals or university memoirs and is listed in the database of the Japanese National Diet Library (Tokyo, Japan).

The search terms were ‘saliva’, ‘amylase’ and ‘exercise’. Original studies published after 2006 concerning the effect of exercise on salivary α-amylase in healthy humans were selected according to the following exclusion criteria: published prior to 2006; the article was not original; the participants were not healthy; the study was intervention-based rather than exercise-based; salivary α-amylase levels/activity were not examined; the language was other than English or Japanese.

**Figure 1:**
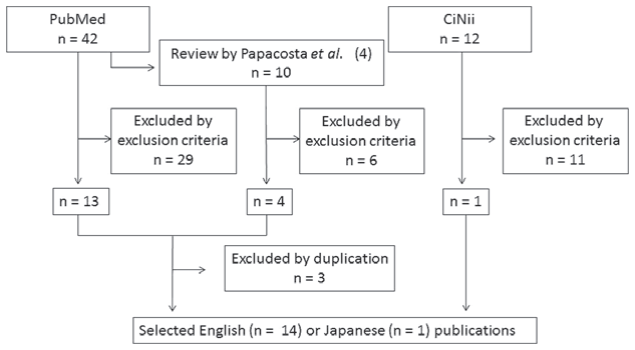
Flow diagram of the literature search and process for selecting original studies that demonstrated a correlation between salivary α-amylase levels and exercise in healthy humans.

The PubMed search identified 42 studies. Fifteen reports were excluded as they were published prior to 2006. Thirteen studies were selected from the titles and abstracts of the remaining 27 publications, according to the aforementioned exclusion criteria. The CiNii search identified 12 studies. One was selected via the same procedure described for the PubMed search. Among the 42 publications obtained from PubMed, one review article by Papacosta and Nassis (5) cited 128 publications. According to the exclusion criteria, four articles were selected from the 10 listed among the references that described the correlation between salivary α-amylase and exercise in healthy humans. Three duplicated studies were excluded and the remaining 15 publications were selected (Fig. 1).

Data are presented as the mean ± SD unless otherwise specified. P<0.05 was considered to indicate a statistically significant difference.

## 3. Results and Discussion

Ten of the 15 publications observed significant increases in salivary α-amylase activity or levels in response to exercise, five identified no differences and no studies identified a reduction (Table I).

A simple comparison or meta-analysis was not applicable as the type, duration and intensity of exercise, and the characteristics of the study subjects differed markedly.

Eight studies defined exercise intensity as a ratio (%) of the maximum or peak oxygen uptake (VO_2_max and VO_2_peak, respectively) or peak power output of the study participants and four used ergometers (6–9) and treadmills (10–13) for exercise loading.

Ergometer exercise was consistently demonstrated to elevate salivary α-amylase activity. Bishop *et al* (6) noted an increase in salivary α-amylase activity following exercise at 70% VO_2_peak for 90 min in endurance-trained males (age 23±1 years; mean ± SEM). Allgrove *et al* (7,8) conducted two studies using a bicycle ergometer, one of which determined the effect of exercise in ten active males (age, 23±1 years; mean ± SEM) at intensities of 50% VO_2_max, 75% VO_2_max and at incremental loads to exhaustion. The duration was matched to the initial VO_2_max test. Levels of α-amylase activity increased in all three trials in response to exercise (7). The other study confirmed these results in 24 trained male participants (age, 23±5 years) who cycled for 2.5 h at 60% VO_2_max followed by 75% VO_2_max to exhaustion; the mean salivary α-amylase activity increased from 143±23 to 463±22 U/ml (8). Fortes *et al* (9) observed an increase (not significant) in salivary α-amylase activity during exercise at 55% peak power output at 33°C, with ≤50% relative humidity; up to 3% of body mass was lost due to sweat in 13 participants (age 24±5 years). The control condition, with rehydration to offset fluid loss, was examined and the kinetics of salivary α-amylase activity were almost identical. The participants in these four studies of ergometer exercise were all healthy, with a mean age of ~23–24 years. The intensity of the exercise was low in the study of dehydration (9). Allgrove *et al* (7) showed that α-amylase activity increased at 50% VO_2_max and the study by Fortes *et al* indicated that the mean α-amylase activity increased at 55% peak power output, although not significantly (9). Thus, exercise on a bicycle ergometer at an intensity as low as 55% peak power output may elevate salivary α-amylase activity.

By contrast, treadmill running generated mixed results. Fortes and Whitham (10) observed that α-amylase activity was elevated following running on a treadmill for 30 min at 50% VO_2_max followed by 30 min at 70% VO_2_max, in six endurance-trained males (age, 21.8±1.9 years). Leicht *et al* (11) reported that α-amylase activity increased in 23 wheelchair athletes. However, subsequent publications did not confirm these results. According to Costa *et al* (12), salivary α-amylase activity increased, although not significantly, in 11 male endurance runners who ran at 75% VO_2_max for 2 h. The findings of Rosa *et al* (13) from a study of 10 active males who ran on treadmills at 70% VO_2_max for 1 h supported these results; the mean salivary α-amylase concentrations were increased but the increase was not statistically significant. Three of the four studies, with the exception of the study of wheelchair athletes, comprised small cohorts, which may account for this discrepancy.

Five studies demonstrated changes in salivary α-amylase in response to exercise without specifying the exercise intensity (14–18). In one of these studies, 12 Caucasian male national-level cyclists underwent a progressive test on a bicycle ergometer. The initial load was 50 W, which increased by 25 W every 2 min to exhaustion. The salivary α-amylase concentration increased in parallel with the increase in load (14). Galina *et al* (15) adopted the Bruce protocol test using treadmills. Twenty-one active males performed a single bout of exercise and a minimum of five stages of the Bruce protocol (19). Salivary α-amylase activity increased during the exercise and reached the greatest level following the highest completed stage achieved by each participant (15). Allgrove *et al* (16) examined responses in male athletes with spinal cord damage. Salivary α-amylase activity increased from 158±47 to 281±72 U/ml (SEM) following 1 h of self-paced handcycling time trials in nine physically active male wheelchair athletes. Ishiguro *et al* (17) observed changes in α-amylase activity among healthy elderly individuals (age 64.7±8.2 years) during a fitness program comprising a 10 min warm up, 30 min of exercise and a 10 min cool down. The exercise performed was light aerobic gymnastics with singing developed for the elderly and the warm up and cool down consisted of stretching. Salivary α-amylase activity were not affected by the program, as pre-exercise values compared with post-exercise values were 32.7±34.0 versus 36.3±34.9 U/ml, respectively. Yamaguchi *et al* (18) identified that levels of salivary α-amylase activity in 10 male university students (age 22.2±0.5 years) during a 20 min walk, in forest and urban environments, did not change. With the exception of light gymnastics for the elderly (17) and relaxed walking (18), physical exercise appears to increase salivary α-amylase activity and concentration (14–16).

Chiodo *et al* (20) and Diaz *et al* (21) investigated the effect of Taekwondo and swimming competitions, respectively. Sixteen taekwondo black belt athletes participated in an official youth competition consisting of three 2-min rounds with 1-min intervals. Salivary α-amylase activity was increased by 115% at the end of the competition compared with the pre-competition values (20). Diaz *et al* (21) compared the α-amylase concentrations in saliva during a national swimming competition with those two weeks following the event (the control day) in 11 professional swimmers. The α-amylase concentrations immediately prior to warming up for the race and 5 min after finishing were higher than those at the same time on the control day. Thus, psychological and physical stress were considered to contribute to the increase in α-amylase levels.

In conclusion, exercise has consistently been shown to increase mean salivary α-amylase activity and concentration in all studies examined in the present review, including those in which changes were not significant, with the exception of the 20-min forest walk (18). The effect tended to be more pronounced at exercise intensities >70% VO_2_max in healthy young individuals. Therefore, studies published following those reviewed by Granger *et al* (1) confirm the conclusion that salivary α-amylase levels markedly increase in response to physical stress. Therefore, α-amylase levels may be an effective non-invasive marker of physical stress.

## Figures and Tables

**Table I:** Parameters of primary studies.

Subjects	Exercise	Changes in salivary α-amylase	Trend of change in salivary α-amylase	Ref.
Endurance-trained males (n=11); age, 23±1 years^a^	Bicycle ergometer 70% VO_2_peak (90 min)	Pre- vs. 45 min following exercise and post-exercise: 441±81 vs. 1279±248 and 1441±262 U/ml^a^	Increase	6
Healthy males (n=10); age, 23±1 years^a^	Bicycle ergometer 50% or 75% VO_2_max or repetition of incremental test to exhaustion (same duration as initial VO_2_max test)	α-amylase activity, mean ± SEM (U/ml): 50% VO_2_max, 450±54→552±77 75% VO_2_max, 372±65→674±77 Exhaustion, 456±65→710±41	Increase	7
Trained male volunteers with cycling as primary sport (n=24); age, 23±5 years	Bicycle ergometer 60% (2.5 h) and 75% VO_2_max to exhaustion	Pre- vs. post-exercise (to exhaustion), 143±23 vs. 463±22 U/ml	Increase	8
Healthy males (n=9), healthy females (n=4); age, 24±5 years	Bicycle ergometer 55% peak power output at 33°C ≤50%RH up to 3% body weight loss as sweat	Exercise tended to increase mean salivary α-amylase activity (NS). Dehydration decreased secretion rate but, did not influence salivary α-amylase activity	No change	9
Healthy endurance-trained males (n=6); age, 21.8±1.9 years	Treadmill running 50% and 70% VO_2_max (30 min each) at 30°C and 40% RH	Pre- vs. post-exercise, 115±27 vs. 180±29 U/ml	Increase	10
Elite male wheelchair athletes (n=23); mean age, 27 years	Treadmill, constant load: 60% VO_2_peak (30 min × 2) Intermittent trial: 20 sets of 2 min at 80% VO_2_peak and 1 min at 40% VO_2_peak	Increased following exercise under constant load and intermittent trial	Increase	11
Male competitive endurance runner (n=11); age, 27±7 years	Treadmill 75%VO_2_max (2 h × 2)	Mean activity elevated but NS	No change	12
Male, habitual exercise ≥3 x/week (n=10); age, 23.5±3.95 years^a^	Treadmill, overnight fast then 70% VO_2_peak 1 h after exercise	Mean salivary α-amylase elevated then leveled marginally but NS	No change	13
Male national-level Caucasian cyclists(n=12); age, 22.62±3.51 years	Cycle ergometer Initial load of 50 W, increased by 25 W every 2 min to exhaustion	Elevated α-amylase concentration in salivary proteins	Increase	14
Active males (n=21); age, 24±2 years	Treadmill 3 min warm-up walk at 0.765 m/sec, single exercise test and ≥5 stages of the Bruce protocol following 1.5 min at peak stage; immediate stop	Pre-exercise vs. stop-point: 45.9±13.7 vs. 279.3±26.7 U/ml	Increase	15
Male paraplegic athletes (n=9); age, 44±2 years^a^	Handcycle Self-paced time trial (1 h)	Pre- vs. post-exercise 158±47 vs. 281±72 U/ml^a^	Increase	16
Healthy elderly males (n=7) and females (n=13); age, 64.7±8.2 years	Fitness program for elderly	Pre- vs. post-exercise (NS) 32.7±34.0 vs. 36.3±34.9 U/ml	No change	17
Male university students; n=10; age, 22.2±0.47 years	Walk (20 min) in a forest or urban environment	Mean activity increased in the urban environment and was unchanged in the forest environment (NS)	No change	18
Black belt taekwondo athletes; male (n=10), 14±0 years^a^; female (n=6), (13±1 years^a^)	Saliva collected pre- and post youth competition	Elevated during competition	Increase	20
Male professional swimmers (n=11); age, 21.5±2.16 years	Saliva samples, collected on the first day of national competition and 2 weeks after	Increased salivary α-amylase levels immediately prior to warming up and at 5 min after competition	Increase	21

Data are presented as the mean ± SD unless otherwise specified.

a Mean ± SEM.

RH, relative humidity; VO_2_max, maximal oxygen consumption; VO_2_peak, peak oxygen consumption at high intensity workload; NS, not significant.
